# Supplementary material for: Micropropagation of pokeweed (Phytolacca americana L.) and comparison of phenolic, flavonoid content, and antioxidant activity between pokeweed callus and other parts
Source: PeerJ. 2022 Feb 7;10:e12892. doi: 10.7717/peerj.12892 (PMC8830332; doi:10.7717/peerj.12892)
Supplement: Supplemental Information 1 [file peerj-10-12892-s001.docx]

**Table 1** Raw data of callus induction of different pokeweed explants affected by various types and concentration of auxins

| Explant types | Auxins  Concentrations (mg/l) | Replications | Callus induction percentage | Fresh weight (g) | Dry weight (g) |
| --- | --- | --- | --- | --- | --- |
| Cotyledons | Control | 1 | 0 | - | - |
|  |  | 2 | 0 | - | - |
|  |  | 3 | 0 | - | - |
|  |  | 4 | 0 | - | - |
|  |  | 5 | 0 | - | - |
|  |  | 6 | 0 | - | - |
|  |  | 7 | 0 | - | - |
|  |  | 8 | 0 | - | - |
|  |  | 9 | - | - | - |
|  |  | 10 | - | - | - |
|  |  | 11 | - | - | - |
|  |  | 12 | - | - | - |
|  |  | 13 | - | - | - |
|  |  | 14 | - | - | - |
|  |  | 15 | - | - | - |
|  |  | 16 | - | - | - |
|  | 1 mg/l  2,4-D | 1 | 100 | 0.5827 | 0.0328 |
|  |  | 2 | 100 | 0.3417 | 0.0264 |
|  |  | 3 | 100 | 0.3706 | 0.0295 |
|  |  | 4 | 100 | 0.4049 | 0.0403 |
|  |  | 5 | 100 | 0.4708 | 0.0264 |
|  |  | 6 | 100 | 0.5212 | 0.0202 |
|  |  | 7 | 100 | 0.3548 | 0.0272 |
|  |  | 8 | 100 | 0.3560 | 0.0386 |
|  |  | 9 | - | 0.5810 | 0.0341 |
|  |  | 10 | - | 0.5159 | 0.0248 |
|  |  | 11 | - | 0.4107 | 0.0238 |
|  |  | 12 | - | 0.3879 | 0.0232 |
|  |  | 13 | - | 0.4200 | 0.0221 |
|  |  | 14 | - | 0.4210 | 0.0255 |
|  |  | 15 | - | 0.5332 | 0.0333 |
|  |  | 16 | - | 0.4361 | 0.0286 |
|  | 2 mg/l  2,4-D | 1 | 100 | 0.5001 | 0.0311 |
|  |  | 2 | 100 | 0.5533 | 0.038 |
|  |  | 3 | 100 | 0.4845 | 0.0328 |
|  |  | 4 | 100 | 0.4922 | 0.0298 |
|  |  | 5 | 100 | 0.3779 | 0.0311 |
|  |  | 6 | 100 | 0.5294 | 0.0276 |
|  |  | 7 | 100 | 0.5214 | 0.0251 |
|  |  | 8 | 100 | 0.4893 | 0.0246 |
|  |  | 9 | - | 0.5653 | 0.0216 |
|  |  | 10 | - | 0.7701 | 0.0224 |
|  |  | 11 | - | 0.4684 | 0.0313 |
|  |  | 12 | - | 0.5954 | 0.033 |
|  |  | 13 | - | 0.5889 | 0.0245 |
|  |  | 14 | - | 0.4982 | 0.029 |
|  |  | 15 | - | 0.5553 | 0.0226 |
|  |  | 16 | - | 0.6222 | 0.0236 |
|  | 3 mg/l  2,4-D | 1 | 100 | 0.5618 | 0.0207 |
|  |  | 2 | 100 | 0.3759 | 0.0301 |
|  |  | 3 | 100 | 0.4804 | 0.0306 |
|  |  | 4 | 100 | 0.3282 | 0.0262 |
|  |  | 5 | 100 | 0.5306 | 0.024 |
|  |  | 6 | 100 | 0.5655 | 0.0278 |
|  |  | 7 | 100 | 0.5624 | 0.035 |
|  |  | 8 | 100 | 0.4549 | 0.0281 |
|  |  | 9 | - | 0.5296 | 0.0289 |
|  |  | 10 | - | 0.6262 | 0.0348 |
|  |  | 11 | - | 0.5423 | 0.0279 |
|  |  | 12 | - | 0.4143 | 0.0345 |
|  |  | 13 | - | 0.4124 | 0.0261 |
|  |  | 14 | - | 0.4315 | 0.0213 |
|  |  | 15 | - | 0.5362 | 0.0173 |
|  |  | 16 | - | 0.6425 | 0.023 |
|  | 4 mg/l  2,4-D | 1 | 100 | 0.6543 | 0.0263 |
|  |  | 2 | 100 | 0.5020 | 0.0298 |
|  |  | 3 | 100 | 0.3879 | 0.0232 |
|  |  | 4 | 100 | 0.5897 | 0.0325 |
|  |  | 5 | 100 | 0.5764 | 0.0221 |
|  |  | 6 | 100 | 0.5285 | 0.0249 |
|  |  | 7 | 100 | 0.5490 | 0.0214 |
|  |  | 8 | 100 | 0.3948 | 0.0365 |
|  |  | 9 | - | 0.5711 | 0.0284 |
|  |  | 10 | - | 0.4887 | 0.0259 |
|  |  | 11 | - | 0.4389 | 0.0246 |
|  |  | 12 | - | 0.5602 | 0.0336 |
|  |  | 13 | - | 0.6400 | 0.0349 |
|  |  | 14 | - | 0.4757 | 0.0307 |
|  |  | 15 | - | 0.4600 | 0.0339 |
|  |  | 16 | - | 0.6380 | 0.0307 |
|  | 1 mg/l  IBA | 1 | 0 | - | - |
|  |  | 2 | 0 | - | - |
|  |  | 3 | 0 | - | - |
|  |  | 4 | 0 | - | - |
|  |  | 5 | 0 | - | - |
|  |  | 6 | 0 | - | - |
|  |  | 7 | 0 | - | - |
|  |  | 8 | 0 | - | - |
|  |  | 9 | - | - | - |
|  |  | 10 | - | - | - |
|  |  | 11 | - | - | - |
|  |  | 12 | - | - | - |
|  |  | 13 | - | - | - |
|  |  | 14 | - | - | - |
|  |  | 15 | - | - | - |
|  |  | 16 | - | - | - |
|  | 2 mg/l  IBA | 1 | 0 | - | - |
|  |  | 2 | 0 | - | - |
|  |  | 3 | 0 | - | - |
|  |  | 4 | 0 | - | - |
|  |  | 5 | 0 | - | - |
|  |  | 6 | 0 | - | - |
|  |  | 7 | 0 | - | - |
|  |  | 8 | 0 | - | - |
|  |  | 9 | - | - | - |
|  |  | 10 | - | - | - |
|  |  | 11 | - | - | - |
|  |  | 12 | - | - | - |
|  |  | 13 | - | - | - |
|  |  | 14 | - | - | - |
|  |  | 15 | - | - | - |
|  |  | 16 | - | - | - |
|  | 3 mg/l  IBA | 1 | 0 | - | - |
|  |  | 2 | 0 | - | - |
|  |  | 3 | 0 | - | - |
|  |  | 4 | 0 | - | - |
|  |  | 5 | 0 | - | - |
|  |  | 6 | 0 | - | - |
|  |  | 7 | 0 | - | - |
|  |  | 8 | 0 | - | - |
|  |  | 9 | - | - | - |
|  |  | 10 | - | - | - |
|  |  | 11 | - | - | - |
|  |  | 12 | - | - | - |
|  |  | 13 | - | - | - |
|  |  | 14 | - | - | - |
|  |  | 15 | - | - | - |
|  |  | 16 | - | - | - |
|  | 4 mg/l  IBA | 1 | 0 | - | - |
|  |  | 2 | 0 | - | - |
|  |  | 3 | 0 | - | - |
|  |  | 4 | 0 | - | - |
|  |  | 5 | 0 | - | - |
|  |  | 6 | 0 | - | - |
|  |  | 7 | 0 | - | - |
|  |  | 8 | 0 | - | - |
|  |  | 9 | - | - | - |
|  |  | 10 | - | - | - |
|  |  | 11 | - | - | - |
|  |  | 12 | - | - | - |
|  |  | 13 | - | - | - |
|  |  | 14 | - | - | - |
|  |  | 15 | - | - | - |
|  |  | 16 | - | - | - |
| Explant types | Auxins  Concentrations (mg/l) | Replications | Callus induction percentage | Fresh weight (g) | Dry weight (g) |
| Leaves | Control | 1 | 0 | - | - |
|  |  | 2 | 0 | - | - |
|  |  | 3 | 0 | - | - |
|  |  | 4 | 0 | - | - |
|  |  | 5 | 0 | - | - |
|  |  | 6 | 0 | - | - |
|  |  | 7 | 0 | - | - |
|  |  | 8 | 0 | - | - |
|  |  | 9 | - | - | - |
|  |  | 10 | - | - | - |
|  |  | 11 | - | - | - |
|  |  | 12 | - | - | - |
|  |  | 13 | - | - | - |
|  |  | 14 | - | - | - |
|  |  | 15 | - | - | - |
|  |  | 16 | - | - | - |
|  | 1 mg/l  2,4-D | 1 | 100 | 0.8000 | 0.0343 |
|  |  | 2 | 100 | 0.7192 | 0.0411 |
|  |  | 3 | 100 | 0.5379 | 0.0408 |
|  |  | 4 | 100 | 0.5366 | 0.0329 |
|  |  | 5 | 100 | 0.7788 | 0.0479 |
|  |  | 6 | 100 | 1.0916 | 0.0362 |
|  |  | 7 | 100 | 0.8035 | 0.0366 |
|  |  | 8 | 100 | 0.8069 | 0.0607 |
|  |  | 9 | - | 0.482 | 0.0316 |
|  |  | 10 | - | 0.6987 | 0.0352 |
|  |  | 11 | - | 0.718 | 0.0478 |
|  |  | 12 | - | 0.5604 | 0.0317 |
|  |  | 13 | - | 0.5122 | 0.0439 |
|  |  | 14 | - | 0.7817 | 0.0416 |
|  |  | 15 | - | 0.6525 | 0.0445 |
|  |  | 16 | - | 0.7221 | 0.0355 |
|  | 2 mg/l  2,4-D | 1 | 100 | 0.8952 | 0.0404 |
|  |  | 2 | 100 | 0.5062 | 0.0439 |
|  |  | 3 | 100 | 0.8743 | 0.0519 |
|  |  | 4 | 100 | 0.9512 | 0.0539 |
|  |  | 5 | 100 | 0.7092 | 0.0449 |
|  |  | 6 | 100 | 0.8201 | 0.0515 |
|  |  | 7 | 100 | 0.8044 | 0.0565 |
|  |  | 8 | 100 | 0.8576 | 0.0403 |
|  |  | 9 | - | 0.9037 | 0.05 |
|  |  | 10 | - | 0.8939 | 0.0492 |
|  |  | 11 | - | 0.6107 | 0.0579 |
|  |  | 12 | - | 0.739 | 0.0448 |
|  |  | 13 | - | 0.8513 | 0.0528 |
|  |  | 14 | - | 0.713 | 0.0547 |
|  |  | 15 | - | 0.992 | 0.0379 |
|  |  | 16 | - | 0.7447 | 0.0386 |
|  | 3 mg/l  2,4-D | 1 | 100 | 0.8911 | 0.0296 |
|  |  | 2 | 100 | 0.9043 | 0.0296 |
|  |  | 3 | 100 | 0.7746 | 0.0356 |
|  |  | 4 | 100 | 0.6372 | 0.0516 |
|  |  | 5 | 100 | 0.7249 | 0.0461 |
|  |  | 6 | 100 | 0.691 | 0.0437 |
|  |  | 7 | 100 | 0.556 | 0.0238 |
|  |  | 8 | 100 | 0.8054 | 0.0413 |
|  |  | 9 | - | 0.3443 | 0.0484 |
|  |  | 10 | - | 0.4009 | 0.0248 |
|  |  | 11 | - | 1.0127 | 0.047 |
|  |  | 12 | - | 0.7762 | 0.0442 |
|  |  | 13 | - | 0.899 | 0.0328 |
|  |  | 14 | - | 0.5067 | 0.0475 |
|  |  | 15 | - | 0.7012 | 0.0355 |
|  |  | 16 | - | 0.5208 | 0.0584 |
|  | 4 mg/l  2,4-D | 1 | 100 | 0.4802 | 0.0461 |
|  |  | 2 | 100 | 0.7516 | 0.0313 |
|  |  | 3 | 100 | 0.8685 | 0.0412 |
|  |  | 4 | 100 | 0.9109 | 0.0483 |
|  |  | 5 | 100 | 0.7473 | 0.0474 |
|  |  | 6 | 100 | 0.4823 | 0.0315 |
|  |  | 7 | 100 | 0.7868 | 0.0456 |
|  |  | 8 | 100 | 0.8618 | 0.0448 |
|  |  | 9 | - | 0.6972 | 0.045 |
|  |  | 10 | - | 0.7786 | 0.0474 |
|  |  | 11 | - | 0.7876 | 0.0339 |
|  |  | 12 | - | 0.5736 | 0.0329 |
|  |  | 13 | - | 0.7328 | 0.0486 |
|  |  | 14 | - | 0.7264 | 0.0496 |
|  |  | 15 | - | 0.8616 | 0.043 |
|  |  | 16 | - | 0.8805 | 0.0476 |
|  | 1 mg/l  IBA | 1 | 0 | - | - |
|  |  | 2 | 0 | - | - |
|  |  | 3 | 0 | - | - |
|  |  | 4 | 0 | - | - |
|  |  | 5 | 0 | - | - |
|  |  | 6 | 0 | - | - |
|  |  | 7 | 0 | - | - |
|  |  | 8 | 0 | - | - |
|  |  | 9 | - | - | - |
|  |  | 10 | - | - | - |
|  |  | 11 | - | - | - |
|  |  | 12 | - | - | - |
|  |  | 13 | - | - | - |
|  |  | 14 | - | - | - |
|  |  | 15 | - | - | - |
|  |  | 16 | - | - | - |
|  | 2 mg/l  IBA | 1 | 0 | - | - |
|  |  | 2 | 0 | - | - |
|  |  | 3 | 0 | - | - |
|  |  | 4 | 0 | - | - |
|  |  | 5 | 0 | - | - |
|  |  | 6 | 0 | - | - |
|  |  | 7 | 0 | - | - |
|  |  | 8 | 0 | - | - |
|  |  | 9 | - | - | - |
|  |  | 10 | - | - | - |
|  |  | 11 | - | - | - |
|  |  | 12 | - | - | - |
|  |  | 13 | - | - | - |
|  |  | 14 | - | - | - |
|  |  | 15 | - | - | - |
|  |  | 16 | - | - | - |
|  | 3 mg/l  IBA | 1 | 0 | - | - |
|  |  | 2 | 0 | - | - |
|  |  | 3 | 0 | - | - |
|  |  | 4 | 0 | - | - |
|  |  | 5 | 0 | - | - |
|  |  | 6 | 0 | - | - |
|  |  | 7 | 0 | - | - |
|  |  | 8 | 0 | - | - |
|  |  | 9 | - | - | - |
|  |  | 10 | - | - | - |
|  |  | 11 | - | - | - |
|  |  | 12 | - | - | - |
|  |  | 13 | - | - | - |
|  |  | 14 | - | - | - |
|  |  | 15 | - | - | - |
|  |  | 16 | - | - | - |
|  | 4 mg/l  IBA | 1 | 0 | - | - |
|  |  | 2 | 0 | - | - |
|  |  | 3 | 0 | - | - |
|  |  | 4 | 0 | - | - |
|  |  | 5 | 0 | - | - |
|  |  | 6 | 0 | - | - |
|  |  | 7 | 0 | - | - |
|  |  | 8 | 0 | - | - |
|  |  | 9 | - | - | - |
|  |  | 10 | - | - | - |
|  |  | 11 | - | - | - |
|  |  | 12 | - | - | - |
|  |  | 13 | - | - | - |
|  |  | 14 | - | - | - |
|  |  | 15 | - | - | - |
|  |  | 16 | - | - | - |
| Explant types | Auxins  Concentrations (mg/l) | Replications | Callus induction percentage | Fresh weight (g) | Dry weight (g) |
| Internodes | Control | 1 | 0 | - | - |
|  |  | 2 | 0 | - | - |
|  |  | 3 | 0 | - | - |
|  |  | 4 | 0 | - | - |
|  |  | 5 | 0 | - | - |
|  |  | 6 | 0 | - | - |
|  |  | 7 | 0 | - | - |
|  |  | 8 | 0 | - | - |
|  |  | 9 | - | - | - |
|  |  | 10 | - | - | - |
|  |  | 11 | - | - | - |
|  |  | 12 | - | - | - |
|  |  | 13 | - | - | - |
|  |  | 14 | - | - | - |
|  |  | 15 | - | - | - |
|  |  | 16 | - | - | - |
|  | 1 mg/l  2,4-D | 1 | 100 | 0.308 | 0.0195 |
|  |  | 2 | 100 | 0.3701 | 0.023 |
|  |  | 3 | 100 | 0.1778 | 0.0141 |
|  |  | 4 | 100 | 0.36 | 0.0235 |
|  |  | 5 | 100 | 0.2044 | 0.013 |
|  |  | 6 | 100 | 0.1893 | 0.015 |
|  |  | 7 | 100 | 0.3711 | 0.0187 |
|  |  | 8 | 100 | 0.2116 | 0.0157 |
|  |  | 9 | - | 0.2107 | 0.013 |
|  |  | 10 | - | 0.1965 | 0.0144 |
|  |  | 11 | - | 0.0767 | 0.0047 |
|  |  | 12 | - | 0.2233 | 0.0143 |
|  |  | 13 | - | 0.158 | 0.011 |
|  |  | 14 | - | 0.0937 | 0.0055 |
|  |  | 15 | - | 0.2376 | 0.0151 |
|  |  | 16 | - | 0.2007 | 0.0143 |
|  | 2 mg/l  2,4-D | 1 | 100 | 0.2031 | 0.014 |
|  |  | 2 | 100 | 0.2211 | 0.0167 |
|  |  | 3 | 100 | 0.2235 | 0.0148 |
|  |  | 4 | 100 | 0.2622 | 0.0177 |
|  |  | 5 | 100 | 0.2258 | 0.0173 |
|  |  | 6 | 100 | 0.161 | 0.0119 |
|  |  | 7 | 100 | 0.1842 | 0.0141 |
|  |  | 8 | 100 | 0.1703 | 0.0143 |
|  |  | 9 | - | 0.1708 | 0.0123 |
|  |  | 10 | - | 0.1333 | 0.0079 |
|  |  | 11 | - | 0.2293 | 0.0142 |
|  |  | 12 | - | 0.2405 | 0.0185 |
|  |  | 13 | - | 0.2098 | 0.0142 |
|  |  | 14 | - | 0.2289 | 0.0166 |
|  |  | 15 | - | 0.1662 | 0.0106 |
|  |  | 16 | - | 0.3739 | 0.0261 |
|  | 3 mg/l  2,4-D | 1 | 100 | 0.1927 | 0.0154 |
|  |  | 2 | 100 | 0.2577 | 0.0192 |
|  |  | 3 | 100 | 0.3038 | 0.0214 |
|  |  | 4 | 100 | 0.4472 | 0.0311 |
|  |  | 5 | 100 | 0.187 | 0.0135 |
|  |  | 6 | 100 | 0.1335 | 0.0093 |
|  |  | 7 | 100 | 0.1962 | 0.0132 |
|  |  | 8 | 100 | 0.1486 | 0.0086 |
|  |  | 9 | - | 0.3339 | 0.0208 |
|  |  | 10 | - | 0.3356 | 0.0236 |
|  |  | 11 | - | 0.1961 | 0.0106 |
|  |  | 12 | - | 0.4099 | 0.0202 |
|  |  | 13 | - | 0.1009 | 0.0071 |
|  |  | 14 | - | 0.2249 | 0.015 |
|  |  | 15 | - | 0.1913 | 0.0096 |
|  |  | 16 | - | 0.1224 | 0.008 |
|  | 4 mg/l  2,4-D | 1 | 100 | 0.2392 | 0.0174 |
|  |  | 2 | 100 | 0.2695 | 0.0127 |
|  |  | 3 | 100 | 0.1932 | 0.0113 |
|  |  | 4 | 100 | 0.2209 | 0.0135 |
|  |  | 5 | 100 | 0.2477 | 0.0174 |
|  |  | 6 | 100 | 0.2856 | 0.0185 |
|  |  | 7 | 100 | 0.0876 | 0.0063 |
|  |  | 8 | 100 | 0.1527 | 0.0109 |
|  |  | 9 | - | 0.2433 | 0.0102 |
|  |  | 10 | - | 0.2691 | 0.0128 |
|  |  | 11 | - | 0.2165 | 0.013 |
|  |  | 12 | - | 0.2178 | 0.0136 |
|  |  | 13 | - | 0.2442 | 0.0157 |
|  |  | 14 | - | 0.2623 | 0.0165 |
|  |  | 15 | - | 0.2116 | 0.0142 |
|  |  | 16 | - | 0.1383 | 0.0089 |
|  | 1 mg/l  IBA | 1 | 50 | 0.2593 | 0.0226 |
|  |  | 2 | 100 | 0.2494 | 0.0194 |
|  |  | 3 | 100 | 0.3167 | 0.0243 |
|  |  | 4 | 100 | 0.3979 | 0.0315 |
|  |  | 5 | 50 | 0.2794 | 0.0231 |
|  |  | 6 | 50 | 0.2484 | 0.0206 |
|  |  | 7 | 0 | 0.1682 | 0.0135 |
|  |  | 8 | 0 | 0.4947 | 0.0312 |
|  |  | 9 | - | - | - |
|  |  | 10 | - | - | - |
|  |  | 11 | - | - | - |
|  |  | 12 | - | - | - |
|  |  | 13 | - | - | - |
|  |  | 14 | - | - | - |
|  |  | 15 | - | - | - |
|  |  | 16 | - | - | - |
|  | 2 mg/l  IBA | 1 | 50 | 0.1652 | 0.0116 |
|  |  | 2 | 100 | 0.3626 | 0.0284 |
|  |  | 3 | 100 | 0.397 | 0.0282 |
|  |  | 4 | 100 | 0.4712 | 0.0361 |
|  |  | 5 | 50 | 0.2325 | 0.0199 |
|  |  | 6 | 50 | 0.5537 | 0.043 |
|  |  | 7 | 50 | 0.3438 | 0.0204 |
|  |  | 8 | 0 | 0.2427 | 0.0204 |
|  |  | 9 | - | 0.3899 | 0.0228 |
|  |  | 10 | - | - | - |
|  |  | 11 | - | - | - |
|  |  | 12 | - | - | - |
|  |  | 13 | - | - | - |
|  |  | 14 | - | - | - |
|  |  | 15 | - | - | - |
|  |  | 16 | - | - | - |
|  | 3 mg/l  IBA | 1 | 50 | 0.3176 | 0.0262 |
|  |  | 2 | 50 | 0.3188 | 0.022 |
|  |  | 3 | 50 | 0.5256 | 0.0418 |
|  |  | 4 | 50 | 0.197 | 0.012 |
|  |  | 5 | 50 | 0.3476 | 0.0264 |
|  |  | 6 | 100 | 0.3907 | 0.0276 |
|  |  | 7 | 100 | 0.2614 | 0.0159 |
|  |  | 8 | 0 | 0.4629 | 0.0291 |
|  |  | 9 | - | - | - |
|  |  | 10 | - | - | - |
|  |  | 11 | - | - | - |
|  |  | 12 | - | - | - |
|  |  | 13 | - | - | - |
|  |  | 14 | - | - | - |
|  |  | 15 | - | - | - |
|  |  | 16 | - | - | - |
|  | 4 mg/l  IBA | 1 | 50 | 0.3605 | 0.0264 |
|  |  | 2 | 50 | 0.5348 | 0.0374 |
|  |  | 3 | 50 | 0.4327 | 0.0295 |
|  |  | 4 | 100 | 0.191 | 0.0137 |
|  |  | 5 | 50 | 0.3929 | 0.0236 |
|  |  | 6 | 0 | - | - |
|  |  | 7 | 0 | - | - |
|  |  | 8 | 0 | - | - |
|  |  | 9 | - | - | - |
|  |  | 10 | - | - | - |
|  |  | 11 | - | - | - |
|  |  | 12 | - | - | - |
|  |  | 13 | - | - | - |
|  |  | 14 | - | - | - |
|  |  | 15 | - | - | - |
|  |  | 16 | - | - | - |
| Explant types | Auxins  concentrations  (mg/l) | Replications | Callus induction percentage | Fresh weight (g) | Dry weight (g) |
| Nodes | Control | 1 | 0 | - | - |
|  |  | 2 | 0 | - | - |
|  |  | 3 | 0 | - | - |
|  |  | 4 | 0 | - | - |
|  |  | 5 | 0 | - | - |
|  |  | 6 | 0 | - | - |
|  |  | 7 | 0 | - | - |
|  |  | 8 | 0 | - | - |
|  |  | 9 | - | - | - |
|  |  | 10 | - | - | - |
|  |  | 11 | - | - | - |
|  |  | 12 | - | - | - |
|  |  | 13 | - | - | - |
|  |  | 14 | - | - | - |
|  |  | 15 | - | - | - |
|  |  | 16 | - | - | - |
|  | 1 mg/l  2,4-D | 1 | 100 | 0.6466 | 0.0307 |
|  |  | 2 | 100 | 0.4763 | 0.0326 |
|  |  | 3 | 100 | 0.5695 | 0.0359 |
|  |  | 4 | 100 | 0.604 | 0.03 |
|  |  | 5 | 100 | 0.7073 | 0.0424 |
|  |  | 6 | 100 | 0.5705 | 0.0336 |
|  |  | 7 | 100 | 1.0815 | 0.0469 |
|  |  | 8 | 100 | 0.4214 | 0.0344 |
|  |  | 9 | - | 0.5861 | 0.0252 |
|  |  | 10 | - | 0.5927 | 0.0369 |
|  |  | 11 | - | 0.5787 | 0.0355 |
|  |  | 12 | - | 0.8263 | 0.0347 |
|  |  | 13 | - | 0.4779 | 0.037 |
|  |  | 14 | - | 0.5509 | 0.0516 |
|  |  | 15 | - | 0.5246 | 0.0355 |
|  |  | 16 | - | 0.6212 | 0.0464 |
|  | 2 mg/l  2,4-D | 1 | 100 | 0.5465 | 0.0319 |
|  |  | 2 | 100 | 0.569 | 0.025 |
|  |  | 3 | 100 | 0.5934 | 0.0419 |
|  |  | 4 | 100 | 0.611 | 0.0277 |
|  |  | 5 | 100 | 0.5608 | 0.0374 |
|  |  | 6 | 100 | 0.6743 | 0.0366 |
|  |  | 7 | 100 | 0.3982 | 0.0383 |
|  |  | 8 | 100 | 0.5961 | 0.0287 |
|  |  | 9 | - | 0.3963 | 0.0409 |
|  |  | 10 | - | 0.4509 | 0.0389 |
|  |  | 11 | - | 0.6229 | 0.039 |
|  |  | 12 | - | 0.5733 | 0.0276 |
|  |  | 13 | - | 0.5543 | 0.0229 |
|  |  | 14 | - | 0.655 | 0.0388 |
|  |  | 15 | - | 0.4939 | 0.0336 |
|  |  | 16 | - | 0.6732 | 0.0413 |
|  | 3 mg/l  2,4-D | 1 | 100 | 0.5425 | 0.0355 |
|  |  | 2 | 100 | 0.5769 | 0.0383 |
|  |  | 3 | 100 | 0.5126 | 0.0448 |
|  |  | 4 | 100 | 0.7149 | 0.0447 |
|  |  | 5 | 100 | 0.5798 | 0.0244 |
|  |  | 6 | 100 | 0.6678 | 0.0317 |
|  |  | 7 | 100 | 0.5316 | 0.0336 |
|  |  | 8 | 100 | 0.5367 | 0.0276 |
|  |  | 9 | - | 0.5131 | 0.0421 |
|  |  | 10 | - | 0.6809 | 0.0275 |
|  |  | 11 | - | 0.5997 | 0.0272 |
|  |  | 12 | - | 0.6396 | 0.0266 |
|  |  | 13 | - | 0.412 | 0.0402 |
|  |  | 14 | - | 0.4189 | 0.0448 |
|  |  | 15 | - | 0.5831 | 0.0232 |
|  |  | 16 | - | 0.522 | 0.0342 |
|  | 4 mg/l  2,4-D | 1 | 100 | 0.4764 | 0.0285 |
|  |  | 2 | 100 | 0.4728 | 0.0284 |
|  |  | 3 | 100 | 0.5094 | 0.0303 |
|  |  | 4 | 100 | 0.563 | 0.0419 |
|  |  | 5 | 100 | 0.6656 | 0.0446 |
|  |  | 6 | 100 | 0.5665 | 0.0266 |
|  |  | 7 | 100 | 0.4551 | 0.0353 |
|  |  | 8 | 100 | 0.6989 | 0.0291 |
|  |  | 9 | - | 0.6465 | 0.0387 |
|  |  | 10 | - | 0.5604 | 0.033 |
|  |  | 11 | - | 0.6072 | 0.0333 |
|  |  | 12 | - | 0.6137 | 0.0327 |
|  |  | 13 | - | 0.6251 | 0.0378 |
|  |  | 14 | - | 0.6743 | 0.0404 |
|  |  | 15 | - | 0.6137 | 0.0446 |
|  |  | 16 | - | 0.4881 | 0.0367 |
|  | 1 mg/l  IBA | 1 | 50 | 0.6276 | 0.0509 |
|  |  | 2 | 50 | 0.5863 | 0.0443 |
|  |  | 3 | 50 | 0.5427 | 0.0566 |
|  |  | 4 | 50 | 0.8172 | 0.064 |
|  |  | 5 | 50 | 0.6566 | 0.0602 |
|  |  | 6 | 100 | 0.6969 | 0.0509 |
|  |  | 7 | 100 | 0.824 | 0.0289 |
|  |  | 8 | 50 | 0.2889 | 0.042 |
|  |  | 9 | - | 0.4307 | 0.0516 |
|  |  | 10 | - | 0.7749 | 0.0234 |
|  |  | 11 | - | - | - |
|  |  | 12 | - | - | - |
|  |  | 13 | - | - | - |
|  |  | 14 | - | - | - |
|  |  | 15 | - | - | - |
|  |  | 16 | - | - | - |
|  | 2 mg/l  IBA | 1 | 50 | 0.669 | 0.0486 |
|  |  | 2 | 50 | 0.6917 | 0.0194 |
|  |  | 3 | 50 | 0.4601 | 0.057 |
|  |  | 4 | 100 | 0.6885 | 0.0602 |
|  |  | 5 | 100 | 0.497 | 0.0506 |
|  |  | 6 | 100 | 0.4903 | 0.0441 |
|  |  | 7 | 100 | 0.756 | 0.0383 |
|  |  | 8 | 100 | 0.6627 | 0.0488 |
|  |  | 9 | - | 0.2723 | 0.0585 |
|  |  | 10 | - | 0.7705 | 0.0495 |
|  |  | 11 | - | 0.6283 | 0.0339 |
|  |  | 12 | - | 0.8568 | 0.0341 |
|  |  | 13 | - | 0.5597 | 0.0336 |
|  |  | 14 | - | - | - |
|  |  | 15 | - | - | - |
|  |  | 16 | - | - | - |
|  | 3 mg/l  IBA | 1 | 50 | 0.9986 | 0.0528 |
|  |  | 2 | 50 | 1.1513 | 0.0501 |
|  |  | 3 | 50 | 0.5693 | 0.0549 |
|  |  | 4 | 50 | 0.7938 | 0.0645 |
|  |  | 5 | 50 | 0.6906 | 0.0595 |
|  |  | 6 | 100 | 1.093 | 0.0784 |
|  |  | 7 | 100 | 0.8106 | 0.0417 |
|  |  | 8 | 50 | 0.745 | 0.0696 |
|  |  | 9 | - | 0.8848 | 0.0493 |
|  |  | 10 | - | 0.9615 | 0.0737 |
|  |  | 11 | - | - | - |
|  |  | 12 | - | - | - |
|  |  | 13 | - | - | - |
|  |  | 14 | - | - | - |
|  |  | 15 | - | - | - |
|  |  | 16 | - | - | - |
|  | 4 mg/l  IBA | 1 | 100 | 0.3023 | 0.0211 |
|  |  | 2 | 100 | 0.3763 | 0.0358 |
|  |  | 3 | 50 | 0.8073 | 0.0289 |
|  |  | 4 | 50 | 0.8008 | 0.0713 |
|  |  | 5 | 50 | 0.555 | 0.0402 |
|  |  | 6 | 50 | 0.8197 | 0.05 |
|  |  | 7 | 50 | 0.5085 | 0.0514 |
|  |  | 8 | 50 | 0.6662 | 0.0304 |
|  |  | 9 | - | 0.4713 | 0.0402 |
|  |  | 10 | - | - | - |
|  |  | 11 | - | - | - |
|  |  | 12 | - | - | - |
|  |  | 13 | - | - | - |
|  |  | 14 | - | - | - |
|  |  | 15 | - | - | - |
|  |  | 16 | - | - | - |
