# Supplementary material for: Micropropagation of pokeweed (Phytolacca americana L.) and comparison of phenolic, flavonoid content, and antioxidant activity between pokeweed callus and other parts
Source: PeerJ. 2022 Feb 7;10:e12892. doi: 10.7717/peerj.12892 (PMC8830332; doi:10.7717/peerj.12892)
Supplement: Supplemental Information 3 [file peerj-10-12892-s003.docx]

**Table 3** Raw data Combined effects of cytokinins and NAA on direct shoot organogenesis from pokeweed nodal explants

| Cytokinin and  NAA conc.  (mg/l) | Rep. | Root percentage (%) | Shoot percentage (%) | Number of shoots | Shoot length (cm) | Number of leaves |
| --- | --- | --- | --- | --- | --- | --- |
| Control | 1 | 0 | 0 | 1.0 | 6.9 | 8.0 |
|  | 2 | 50 | 50 | 1.0 | 5.4 | 7.0 |
|  | 3 | 50 | 50 | 1.0 | 6.8 | 8.0 |
|  | 4 | 50 | 50 | 1.0 | 1.8 | 5.0 |
|  | 5 | 0 | 50 | 1.0 | 6.2 | 5.0 |
|  | 6 | 50 | 50 | 1.0 | 6.0 | 7.0 |
|  | 7 | 50 | 50 | 1.0 | 0.7 | 1.0 |
|  | 8 | 0 | 0 | 1.0 | 2.3 | 5.0 |
|  | 9 | - | - | 1.0 | 5.1 | 8.0 |
|  | 10 | - | - | 1.0 | 1.1 | 3.0 |
|  | 11 | - | - | 1.0 | 5.9 | 8.0 |
|  | 12 | - | - | 1.0 | 5.6 | 8.0 |
|  | 13 | - | - | 1.0 | 3.6 | 6.0 |
|  | 14 | - | - | - | - | - |
|  | 15 | - | - | - | - | - |
|  | 16 | - | - | - | - | - |
| 1 mg/l  BAP | 1 | 0 | 100 | 2.0 | 4.4 | 12.0 |
|  | 2 | 0 | 100 | 3.0 | 4.0 | 10.0 |
|  | 3 | 0 | 100 | 1.0 | 5.3 | 9.0 |
|  | 4 | 0 | 100 | 2.0 | 4.6 | 10.0 |
|  | 5 | 0 | 100 | 1.0 | 4.8 | 13.0 |
|  | 6 | 0 | 100 | 3.0 | 4.3 | 9.0 |
|  | 7 | 0 | 100 | 2.0 | 4.6 | 8.0 |
|  | 8 | 0 | 100 | 3.0 | 3.8 | 9.0 |
|  | 9 | - | - | 2.0 | 3.0 | 8.0 |
|  | 10 | - | - | 2.0 | 4.1 | 10.0 |
|  | 11 | - | - | 2.0 | 3.9 | 9.0 |
|  | 12 | - | - | 3.0 | 4.5 | 9.0 |
|  | 13 | - | - | 2.0 | 3.6 | 9.0 |
|  | 14 | - | - | 3.0 | 3.7 | 7.0 |
|  | 15 | - | - | 2.0 | 4.1 | 9.0 |
|  | 16 | - | - | 2.0 | 4.2 | 11.0 |
| 1 mg/l  BAP  +  0.1 mg/l NAA | 1 | 0 | 100 | 2.0 | 4.4 | 6.0 |
|  | 2 | 0 | 100 | 2.0 | 3.5 | 7.0 |
|  | 3 | 0 | 100 | 1.0 | 4.8 | 11.0 |
|  | 4 | 0 | 100 | 1.0 | 3.2 | 6.0 |
|  | 5 | 0 | 100 | 2.0 | 4.2 | 9.0 |
|  | 6 | 0 | 100 | 2.0 | 3.2 | 8.0 |
|  | 7 | 0 | 100 | 2.0 | 3.7 | 6.0 |
|  | 8 | 0 | 100 | 2.0 | 4.1 | 5.0 |
|  | 9 | - | - | 3.0 | 3.9 | 7.0 |
|  | 10 | - | - | 3.0 | 3.5 | 8.0 |
|  | 11 | - | - | 3.0 | 4.2 | 5.0 |
|  | 12 | - | - | 1.0 | 3.8 | 10.0 |
|  | 13 | - | - | 3.0 | 4.0 | 8.0 |
|  | 14 | - | - | 2.0 | 4.3 | 4.0 |
|  | 15 | - | - | 3.0 | 3.5 | 6.0 |
|  | 16 | - | - | 1.0 | 3.6 | 10.0 |
| 1 mg/l  BAP  +  0.2 mg/l NAA | 1 | 0 | 100 | 1.0 | 3.8 | 8.0 |
|  | 2 | 0 | 100 | 1.0 | 4.2 | 5.0 |
|  | 3 | 0 | 100 | 1.0 | 3.3 | 9.0 |
|  | 4 | 0 | 100 | 2.0 | 4.3 | 6.0 |
|  | 5 | 0 | 100 | 2.0 | 3.8 | 5.0 |
|  | 6 | 0 | 100 | 2.0 | 4.0 | 8.0 |
|  | 7 | 0 | 100 | 1.0 | 4.1 | 8.0 |
|  | 8 | 0 | 100 | 3.0 | 3.4 | 7.0 |
|  | 9 | - | - | 1.0 | 3.2 | 8.0 |
|  | 10 | - | - | 2.0 | 4.6 | 5.0 |
|  | 11 | - | - | 1.0 | 4.6 | 6.0 |
|  | 12 | - | - | 3.0 | 3.6 | 5.0 |
|  | 13 | - | - | 2.0 | 3.4 | 6.0 |
|  | 14 | - | - | 2.0 | 3.6 | 6.0 |
|  | 15 | - | - | 2.0 | 3.1 | 5.0 |
|  | 16 | - | - | 1.0 | 3.8 | 6.0 |
| 1 mg/l  BAP  +  0.3 mg/l NAA | 1 | 0 | 100 | 2.0 | 3.8 | 6.0 |
|  | 2 | 0 | 100 | 1.0 | 2.0 | 7.0 |
|  | 3 | 0 | 100 | 3.0 | 3.4 | 8.0 |
|  | 4 | 0 | 100 | 2.0 | 2.5 | 7.0 |
|  | 5 | 0 | 100 | 2.0 | 4.2 | 7.0 |
|  | 6 | 0 | 100 | 1.0 | 3.5 | 8.0 |
|  | 7 | 0 | 100 | 1.0 | 2.4 | 6.0 |
|  | 8 | 0 | 100 | 1.0 | 2.1 | 6.0 |
|  | 9 | - | - | 2.0 | 2.5 | 7.0 |
|  | 10 | - | - | 1.0 | 3.0 | 5.0 |
|  | 11 | - | - | 1.0 | 4.2 | 9.0 |
|  | 12 | - | - | 1.0 | 3.6 | 7.0 |
|  | 13 | - | - | 1.0 | 3.8 | 10.0 |
|  | 14 | - | - | 1.0 | 3.1 | 7.0 |
|  | 15 | - | - | 1.0 | 4.0 | 10.0 |
|  | 16 | - | - | 2.0 | 2.8 | 4.0 |
| 2 mg/l  KIN | 1 | 0 | 100 | 2.0 | 5.5 | 10.0 |
|  | 2 | 0 | 100 | 2.0 | 5.6 | 9.0 |
|  | 3 | 0 | 100 | 1.0 | 5.5 | 14.0 |
|  | 4 | 0 | 100 | 2.0 | 3.6 | 14.0 |
|  | 5 | 0 | 100 | 2.0 | 6.4 | 13.0 |
|  | 6 | 0 | 100 | 2.0 | 4.7 | 15.0 |
|  | 7 | 0 | 100 | 2.0 | 6.7 | 12.0 |
|  | 8 | 0 | 100 | 3.0 | 5.6 | 8.0 |
|  | 9 | - | - | 2.0 | 4.7 | 7.0 |
|  | 10 | - | - | 1.0 | 5.2 | 5.0 |
|  | 11 | - | - | 1.0 | 4.0 | 10.0 |
|  | 12 | - | - | 1.0 | 4.5 | 11.0 |
|  | 13 | - | - | 1.0 | 4.0 | 14.0 |
|  | 14 | - | - | 2.0 | 5.0 | 10.0 |
|  | 15 | - | - | 2.0 | 3.9 | 10.0 |
|  | 16 | - | - | 3.0 | 5.8 | 13.0 |
| 2 mg/l  KIN  +  0.1 mg/l NAA | 1 | 0 | 100 | 1.0 | 4.2 | 5.0 |
|  | 2 | 0 | 100 | 4.0 | 3.5 | 5.0 |
|  | 3 | 0 | 100 | 3.0 | 3.3 | 8.0 |
|  | 4 | 0 | 100 | 2.0 | 3.1 | 3.0 |
|  | 5 | 0 | 100 | 1.0 | 3.0 | 4.0 |
|  | 6 | 0 | 100 | 4.0 | 2.8 | 4.0 |
|  | 7 | 0 | 100 | 1.0 | 3.4 | 9.0 |
|  | 8 | 0 | 100 | 1.0 | 1.5 | 2.0 |
|  | 9 | - | - | 1.0 | 2.4 | 6.0 |
|  | 10 | - | - | 3.0 | 4.3 | 6.0 |
|  | 11 | - | - | 4.0 | 3.7 | 5.0 |
|  | 12 | - | - | 4.0 | 2.8 | 5.0 |
|  | 13 | - | - | 3.0 | 2.0 | 5.0 |
|  | 14 | - | - | 4.0 | 3.5 | 4.0 |
|  | 15 | - | - | 1.0 | 3.0 | 7.0 |
|  | 16 | - | - | 3.0 | 4.2 | 6.0 |
| 2 mg/l  KIN  +  0.2 mg/l NAA | 1 | 0 | 100 | 3.0 | 3.0 | 6.0 |
|  | 2 | 0 | 100 | 1.0 | 4.3 | 6.0 |
|  | 3 | 0 | 100 | 1.0 | 3.7 | 10.0 |
|  | 4 | 0 | 100 | 4.0 | 3.3 | 4.0 |
|  | 5 | 0 | 100 | 3.0 | 3.2 | 5.0 |
|  | 6 | 0 | 100 | 2.0 | 3.5 | 5.0 |
|  | 7 | 0 | 100 | 3.0 | 2.9 | 4.0 |
|  | 8 | 0 | 100 | 2.0 | 4.1 | 8.0 |
|  | 9 | - | - | 2.0 | 4.0 | 6.0 |
|  | 10 | - | - | 4.0 | 2.6 | 5.0 |
|  | 11 | - | - | 1.0 | 0.9 | 2.0 |
|  | 12 | - | - | 3.0 | 2.9 | 4.0 |
|  | 13 | - | - | 5.0 | 2.9 | 5.0 |
|  | 14 | - | - | 1.0 | 0.5 | 2.0 |
|  | 15 | - | - | 3.0 | 3.5 | 6.0 |
|  | 16 | - | - | 2.0 | 2.5 | 6.0 |
| 2 mg/l  KIN  +  0.3 mg/l NAA | 1 | 0 | 100 | 3.0 | 3.4 | 8.0 |
|  | 2 | 0 | 100 | 1.0 | 1.0 | 3.0 |
|  | 3 | 0 | 100 | 2.0 | 2.9 | 6.0 |
|  | 4 | 0 | 100 | 2.0 | 3.5 | 10.0 |
|  | 5 | 0 | 100 | 1.0 | 4.1 | 11.0 |
|  | 6 | 0 | 100 | 2.0 | 3.0 | 6.0 |
|  | 7 | 0 | 100 | 1.0 | 3.6 | 9.0 |
|  | 8 | 0 | 100 | 2.0 | 2.8 | 5.0 |
|  | 9 | - | - | 1.0 | 4.0 | 12.0 |
|  | 10 | - | - | 4.0 | 2.3 | 3.0 |
|  | 11 | - | - | 2.0 | 1.5 | 5.0 |
|  | 12 | - | - | 2.0 | 1.8 | 5.0 |
|  | 13 | - | - | 3.0 | 2.9 | 7.0 |
|  | 14 | - | - | 1.0 | 3.0 | 8.0 |
|  | 15 | - | - | 2.0 | 3.4 | 6.0 |
|  | 16 | - | - | 2.0 | 1.5 | 5.0 |
| 1 mg/l  TDZ | 1 | 0 | 100 | 3.0 | 1.8 | 8.0 |
|  | 2 | 0 | 100 | 2.0 | 3.5 | 12.0 |
|  | 3 | 0 | 100 | 1.0 | 2.8 | 5.0 |
|  | 4 | 0 | 100 | 2.0 | 3.3 | 5.0 |
|  | 5 | 0 | 100 | 1.0 | 4.4 | 9.0 |
|  | 6 | 0 | 100 | 1.0 | 2.1 | 3.0 |
|  | 7 | 0 | 100 | 2.0 | 4.1 | 7.0 |
|  | 8 | 0 | 100 | 3.0 | 3.1 | 5.0 |
|  | 9 | - | - | 4.0 | 3.6 | 9.0 |
|  | 10 | - | - | 2.0 | 3.4 | 9.0 |
|  | 11 | - | - | 2.0 | 1.7 | 10.0 |
|  | 12 | - | - | 4.0 | 2.7 | 7.0 |
|  | 13 | - | - | 3.0 | 4.8 | 7.0 |
|  | 14 | - | - | 4.0 | 2.9 | 5.0 |
|  | 15 | - | - | 3.0 | 2.6 | 8.0 |
|  | 16 | - | - | 2.0 | 3.2 | 6.0 |
| 1 mg/l  TDZ  +  0.1 mg/l NAA | 1 | 0 | 100 | 4.0 | 3.5 | 6.0 |
|  | 2 | 0 | 100 | 3.0 | 2.5 | 7.0 |
|  | 3 | 0 | 100 | 1.0 | 3.0 | 7.0 |
|  | 4 | 0 | 100 | 3.0 | 3.5 | 8.0 |
|  | 5 | 0 | 100 | 1.0 | 2.9 | 6.0 |
|  | 6 | 0 | 100 | 2.0 | 2.5 | 7.0 |
|  | 7 | 0 | 100 | 2.0 | 2.8 | 9.0 |
|  | 8 | 0 | 100 | 1.0 | 4.0 | 9.0 |
|  | 9 | - | - | 1.0 | 3.2 | 7.0 |
|  | 10 | - | - | 3.0 | 3.0 | 5.0 |
|  | 11 | - | - | 4.0 | 4.2 | 8.0 |
|  | 12 | - | - | 4.0 | 4.3 | 8.0 |
|  | 13 | - | - | 3.0 | 3.0 | 6.0 |
|  | 14 | - | - | 3.0 | 3.7 | 8.0 |
|  | 15 | - | - | 1.0 | 1.5 | 7.0 |
|  | 16 | - | - | 2.0 | 3.9 | 9.0 |
| 1 mg/l  TDZ  +  0.2 mg/l NAA | 1 | 0 | 100 | 1.0 | 3.0 | 6.0 |
|  | 2 | 0 | 100 | 1.0 | 3.5 | 6.0 |
|  | 3 | 0 | 100 | 1.0 | 2.2 | 9.0 |
|  | 4 | 0 | 100 | 1.0 | 2.1 | 8.0 |
|  | 5 | 0 | 100 | 1.0 | 1.5 | 5.0 |
|  | 6 | 0 | 100 | 1.0 | 2.0 | 7.0 |
|  | 7 | 0 | 100 | 2.0 | 2.9 | 5.0 |
|  | 8 | 0 | 100 | 2.0 | 3.8 | 6.0 |
|  | 9 | - | - | 4.0 | 2.3 | 4.0 |
|  | 10 | - | - | 1.0 | 3.0 | 7.0 |
|  | 11 | - | - | 1.0 | 2.9 | 6.0 |
|  | 12 | - | - | 1.0 | 1.7 | 4.0 |
|  | 13 | - | - | 2.0 | 2.2 | 7.0 |
|  | 14 | - | - | 4.0 | 3.0 | 5.0 |
|  | 15 | - | - | 3.0 | 2.9 | 5.0 |
|  | 16 | - | - | 1.7 | 2.6 | 6.0 |
| 1 mg/l  TDZ  +  0.3 mg/l NAA | 1 | 0 | 100 | 1.0 | 2.5 | 7.0 |
|  | 2 | 0 | 100 | 1.0 | 4.1 | 11.0 |
|  | 3 | 0 | 100 | 4.0 | 3.1 | 7.0 |
|  | 4 | 0 | 100 | 1.0 | 1.5 | 5.0 |
|  | 5 | 0 | 100 | 1.0 | 1.6 | 3.0 |
|  | 6 | 0 | 100 | 2.0 | 2.7 | 6.0 |
|  | 7 | 0 | 100 | 3.0 | 4.0 | 8.0 |
|  | 8 | 0 | 100 | 1.0 | 2.5 | 6.0 |
|  | 9 | - | - | 3.0 | 3.2 | 5.0 |
|  | 10 | - | - | 2.0 | 3.1 | 7.0 |
|  | 11 | - | - | 1.0 | 2.9 | 6.0 |
|  | 12 | - | - | 2.0 | 2.6 | 6.0 |
|  | 13 | - | - | 1.0 | 3.5 | 7.0 |
|  | 14 | - | - | 1.0 | 3.9 | 8.0 |
|  | 15 | - | - | 2.0 | 3.5 | 7.0 |
|  | 16 | - | - | 1.0 | 2.7 | 7.0 |
