# Supplementary material for: Micropropagation of pokeweed (Phytolacca americana L.) and comparison of phenolic, flavonoid content, and antioxidant activity between pokeweed callus and other parts
Source: PeerJ. 2022 Feb 7;10:e12892. doi: 10.7717/peerj.12892 (PMC8830332; doi:10.7717/peerj.12892)
Supplement: Supplemental Information 4 [file peerj-10-12892-s004.docx]

**Table 4** Raw data of TPC, TFC, and IC50 by DPPH assay of different types of pokeweed explants

| Explant types | Replications | TPC  (mg GAE/g extract) | TFC  (mg QE/g extract) | IC50 by DPPH assay (µg/ml) |
| --- | --- | --- | --- | --- |
| Callus | 1 | 24.90 | 11.67 | 349.36 |
|  | 2 | 26.10 | 12.63 | 364.15 |
|  | 3 | 27.10 | 10.90 | 312.47 |
| Leaf | 1 | 27.90 | 58.59 | 236.57 |
|  | 2 | 29.30 | 57.25 | 222.91 |
|  | 3 | 28.50 | 56.09 | 226.64 |
| Root | 1 | 13.50 | 30.90 | 1136.38 |
|  | 2 | 14.70 | 34.75 | 1156.28 |
|  | 3 | 13.90 | 34.75 | 1083.87 |
| Seed | 1 | 140.30 | 47.25 | 13.75 |
|  | 2 | 156.90 | 50.52 | 12.69 |
|  | 3 | 170.30 | 51.67 | 11.55 |
| Ascorbic acid | 1 | - | - | 3.78 |
|  | 2 | - | - | 3.97 |
|  | 3 | - | - | 3.98 |
