# Supplementary material for: Micropropagation of pokeweed (Phytolacca americana L.) and comparison of phenolic, flavonoid content, and antioxidant activity between pokeweed callus and other parts
Source: PeerJ. 2022 Feb 7;10:e12892. doi: 10.7717/peerj.12892 (PMC8830332; doi:10.7717/peerj.12892)
Supplement: Supplemental Information 5 — Combination effects of cytokinins and NAA on direct shoot organogenesis from pokeweed nodal explants. [file peerj-10-12892-s005.docx]

Combination effects of cytokinins and NAA on direct shoot organogenesis from pokeweed nodal explants

| PGRs | | Roots | Shoots | No. of shoots | Shoot length | No. of leaves |
| --- | --- | --- | --- | --- | --- | --- |
| Cytokinins | NAA  conc. (mg/l) |  |  |  |  |  |
| Control | | 31.25 ± 9.15 *a* | 37.50 ± 8.18 *b* | 1.0000 ± 0.0000 *e* | 4.4125 ± 0.4974 *ab* | 6.0625 ± 0.4956 *cd* |
| 1 mg/l BAP | 0 | 0.00 ± 0.00 *b* | 100.00 ± 0.00 *a* | 2.1875 ± 0.1638 *a-c* | 4.1813 ± 0.1352 *b* | 9.5000 ± 0.3764 *b* |
|  | 0.1 | 0.00 ± 0.00 *b* | 100.00 ± 0.00 *a* | 2.0625 ± 0.1930 *a-d* | 3.8687 ± 0.1124 *b* | 7.2500 ± 0.5041 *c* |
|  | 0.2 | 0.00 ± 0.00 *b* | 100.00 ± 0.00 *a* | 1.6875 ± 0.1760 *cd* | 3.8000 ± 0.1176 *b* | 6.4375 ± 0.3412 *cd* |
|  | 0.3 | 0.00 ± 0.00 *b* | 100.00 ± 0.00 *a* | 1.4375 ± 0.1573 *de* | 3.1813 ± 0.1840 *cd* | 7.1250 ± 0.4070 *c* |
| 2 mg/l KIN | 0 | 0.00 ± 0.00 *b* | 100.00 ± 0.00 *a* | 1.8125 ± 0.1638 *b-d* | 5.0438 ± 0.2266 *a* | 10.9380 ± 0.7099 *a* |
|  | 0.1 | 0.00 ± 0.00 *b* | 100.00 ± 0.00 *a* | 2.5000 ± 0.3291 *a* | 3.1688 ± 0.1942 *cd* | 5.2500 ± 0.4425 *d* |
|  | 0.2 | 0.00 ± 0.00 *b* | 100.00 ± 0.00 *a* | 2.5000 ± 0.3028 *a* | 2.9875 ± 0.2591 *d* | 5.2500 ± 0.4958 *d* |
|  | 0.3 | 0.00 ± 0.00 *b* | 100.00 ± 0.00 *a* | 1.9375 ± 0.2135 *a-d* | 2.7937 ± 0.2323 *d* | 6.8125 ± 0.6660 *c* |
| 1 mg/l TDZ | 0 | 0.00 ± 0.00 *b* | 100.00 ± 0.00 *a* | 2.4375 ± 0.2577 *ab* | 3.1250 ± 0.2171 *d* | 7.1875 ± 0.5789 *c* |
|  | 0.1 | 0.00 ± 0.00 *b* | 100.00 ± 0.00 *a* | 2.3750 ± 0.2869 *ab* | 3.2188 ± 0.1826 *cd* | 7.3125 ± 0.2989 *c* |
|  | 0.2 | 0.00 ± 0.00 *b* | 100.00 ± 0.00 *a* | 1.7313 ± 0.2656 *cd* | 2.6000 ± 0.1586 *d* | 6.0000 ± 0.3416 *cd* |
|  | 0.3 | 0.00 ± 0.00 *b* | 100.00 ± 0.00 *a* | 1.6875 ± 0.2366 *cd* | 2.9625 ± 0.1895 *d* | 6.6250 ± 0.4270 *c* |

Means ± SE followed by the different letter are significantly different according to ANOVA and Least Significant Different Test (*p < 0.05*) The different letters (*a*, *b*, *c…*) are significantly different in the columns (*p* < 0.05).
